# Blue organic light-emitting diodes realizing external quantum efficiency over 25% using thermally activated delayed fluorescence emitters

**DOI:** 10.1038/s41598-017-00368-5

**Published:** 2017-03-21

**Authors:** Takuya Miwa, Shosei Kubo, Katsuyuki Shizu, Takeshi Komino, Chihaya Adachi, Hironori Kaji

**Affiliations:** 1 Institute for Chemical Research, Kyoto University, Uji, Kyoto 611-0011 Japan; 2 Center for Organic Photonics and Electronics Research (OPERA), Kyushu University, 744 Motooka, Nishi, Fukuoka 819-0395 Japan; 3 JST, ERATO, Adachi Molecular Exciton Engineering Project, 744 Motooka, Nishi, Fukuoka 819-0395 Japan

## Abstract

Improving the performance of blue organic light-emitting diodes (OLEDs) is needed for full-colour flat-panel displays and solid-state lighting sources. The use of thermally activated delayed fluorescence (TADF) is a promising approach to efficient blue electroluminescence. However, the difficulty of developing efficient blue TADF emitters lies in finding a molecular structure that simultaneously incorporates (i) a small energy difference between the lowest excited singlet state (S_1_) and the lowest triplet state (T_1_), Δ*E*_ST_, (ii) a large oscillator strength, *f*, between S_1_ and the ground state (S_0_), and (iii) S_1_ energy sufficiently high for blue emission. In this study, we develop TADF emitters named CCX-I and CCX-II satisfying the above requirements. They show blue photoluminescence and high triplet-to-singlet up-conversion yield. In addition, their transition dipole moments are horizontally oriented, resulting in further increase of their electroluminescence efficiency. Using CCX-II as an emitting dopant, we achieve a blue OLED showing a high external quantum efficiency of 25.9%, which is one of the highest EQEs in blue OLEDs reported previously.

## Introduction

Organic light-emitting diodes (OLEDs) are now used commercially in full-colour displays for smartphones and TVs and have great potential to provide energy-savings for applications over large-areas in solid-state lighting sources and for flexible flat-panel displays^1–3^. Great improvements to the electroluminescence efficiency of OLEDs have been achieved by harnessing phosphorescence^4, 5^, triplet-triplet annihilation^6^, and thermally activated delayed fluorescence (TADF)^7, 8^. An internal quantum efficiency of 100% has been realized for green TADF OLEDs^9^. However, it remains challenging to develop highly efficient blue OLEDs. Recently, TADF materials have attracted considerable attention because of their potential for converting all electrogenerated singlet and triplet excitons into light^8^. Since 2012, the luminescence efficiency of blue-to-green TADF emitters has been significantly improved^9–37^. The external quantum efficiency (*η*_EQE_) of sky-blue^14^ and green^9, 38, 39^ TADF-based OLEDs has been increased to 30%. Nevertheless, there have been few studies describing highly efficient blue TADF emitters with *η*_EQE_ over 20%^10–13^.

The *η*_EQE_ of an OLED can be written as *η*_EQE_ = IQE × *η*_out_, where IQE and *η*_out_ are the device’s internal quantum efficiency and light out-coupling factor, respectively. For a TADF-based OLED,

1 $${\rm{I}}{\rm{Q}}{\rm{E}}=[0.25{{\Phi }}_{{\rm{p}}}+\{0.75+0.25(1-{{\Phi }}_{{\rm{p}}})\}\frac{{{\Phi }}_{{\rm{d}}}}{1-{{\Phi }}_{{\rm{p}}}}]\gamma ,$$

where *γ* is the carrier balance ratio of holes and electrons, and *Φ*_p_ and *Φ*_d_ are contributions from prompt fluorescence and delayed fluorescence to the photoluminescence quantum yield (*Φ*_PL_), respectively: *Φ*_PL_ = *Φ*_p_ + *Φ*_d_^40^. The *Φ*_PL_ of a TADF emitter can be increased by reducing the energy difference Δ*E*_ST_ between the lowest excited singlet state (S_1_) and the lowest triplet state (T_1_), and simultaneously increasing the oscillator strength (*f*) between the S_1_ and ground state (S_0_)^25, 41^. A small Δ*E*_ST_ and large *f* are satisfied when the highest occupied molecular orbital (HOMO) and lowest unoccupied molecular orbital (LUMO) of a TADF emitter are moderately separated in space. This HOMO-LUMO separation can be realized in TADF emitters containing covalently linked electron-donating and accepting units^9, 25, 41^. As well as a small Δ*E*_ST_ and large *f*, blue TADF emitters have the additional requirement of a high S_1_ energy. These three requirements limit the choice of electron-donating and accepting units, making it difficult to achieve blue TADF-based OLEDs with a high IQE.

In this study, we developed highly efficient blue TADF emitters, 3-(9*H*-[3,9′-bicarbazol]-9-yl)-9*H*-xanthen-9-one (CCX-I) and 3-(9′*H*-[9,3′:6′,9″-tercarbazol]-9′-yl)-9*H*-xanthen-9-one (CCX-II), simultaneously satisfying (i) a small Δ*E*_ST_, (ii) a large *f*, and (iii) a high S_1_ energy. Both CCX-I and CCX-II show high blue photoluminescence and high triplet-to-singlet conversion efficiency. Furthermore, CCX-I and CCX-II orient parallel to the glass substrate. These features enhance the *η*_EQE_ of CCX-I- and CCX-II-based OLEDs. A CCX-II-based OLED shows a maximum *η*_EQE_ of 25.9% with Commision International de l’Eclairage (CIE) coordinates of (0.15, 0.22). This performance is comparable to that of a blue phosphorescent OLED based on bis(4′,6′-difluorophenylpyridinato)iridium(III) tetrakis(1-pyrazolyl)borate (FIr6) as an emitting dopant^42^ [*η*_EQE_ of 25% and CIE coordinates of (0.14, 0.23)]. With an out-coupling sheet, the maximum *η*_EQE_ of our devices increased to 33.3%.

**Figure 1:**
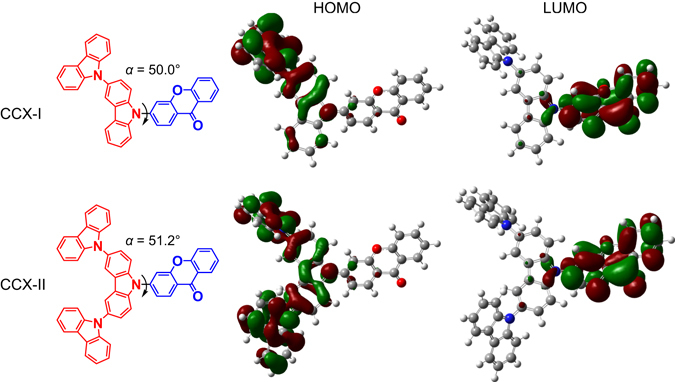
CCX-I and CCX-II. Electron-donating and electron-accepting units are indicated by red and blue, respectively. *α* is the torsion angle between the electron-donating and electron-accepting units. The isosurface value for the HOMOs and LUMOs is 0.02 a.u.

We chose a carbazole derivative with a deep HOMO level as a donor moiety and xanthone with a shallow LUMO level as an acceptor moiety. Figure 1 shows the molecular structures of CCX-I and CCX-II. Quantum chemical calculations were performed with density functional theory (DFT) implemented in the Gaussian 09 program package^43^. Geometry optimization of S_0_ of CCX-I and CCX-II was performed at the PBE0/6-31G(d) level of theory. The *f* and excitation energies for S_1_ and T_1_ were calculated by the time-dependent DFT method implemented in Gaussian 09. Δ*E*_ST_ was calculated as the difference between the S_1_ and T_1_ excitation energies. The HOMOs and LUMOs of the geometry-optimized CCX-I and CCX-II were predominantly distributed over the electron-donating and electron-accepting units, respectively, and were well separated spatially, as shown in Fig. 1. The torsion angles (*α*) between the electron-donating and electron-accepting units of CCX-I and CCX-II were calculated to be 50.0° and 51.2°, respectively. These angles allow for moderate HOMO-LUMO overlap. As shown in Table 1, the calculated Δ*E*_ST_ of CCX-I and CCX-II are smaller than that of 2,4,5,6-tetra(9*H*-carbazol-9-yl)isophthalonitrile (4CzIPN)^8^, one of the most efficient TADF materials developed to date. In addition, the calculated *f* and S_0_-S_1_ excitation energies of CCX-I and CCX-II are larger than that of 4CzIPN, indicating that CCX-I and CCX-II may be efficient blue TADF materials.

**Table 1:** Calculated electronic properties.

Emitter	HOMO (eV)	LUMO (eV)	HOMO-LUMO gap (eV)	*f* ^a^	S_1_^b^ (eV)	T_1_^c^ (eV)	Δ*E*_ST_^d^ (eV)
CCX-I	−5.49	−1.93	3.55	0.1076	2.76	2.71	0.050
CCX-II	−5.50	−2.03	3.47	0.1292	2.73	2.68	0.050
4CzIPN	−5.97	−2.45	3.52	0.0375	2.45	2.26	0.185

^a^Oscillator strength for the S_0_ → S_1_ transition.

^b^S_0_-S_1_ excitation energy.

^c^S_0_-T_1_ excitation energy.

^d^Energy difference between S_1_ and T_1_.

**Figure 2:**
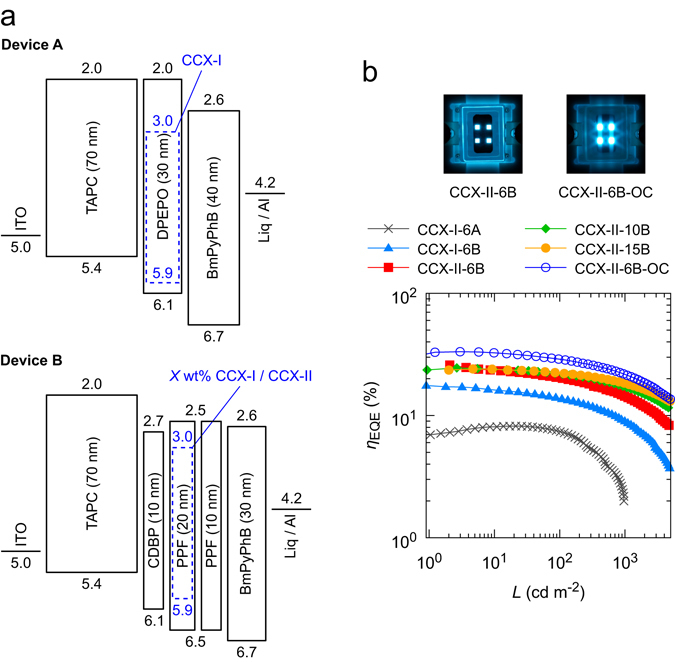
Energy level diagram of the materials for devices and their performance in OLEDs. (**a**) Energy level diagrams of the materials used in the devices. (**b**) *η*_EQE_-*L* characteristics and photos of CCX-II-6B and CCX-II-6B-OC.

**Figure 3:**
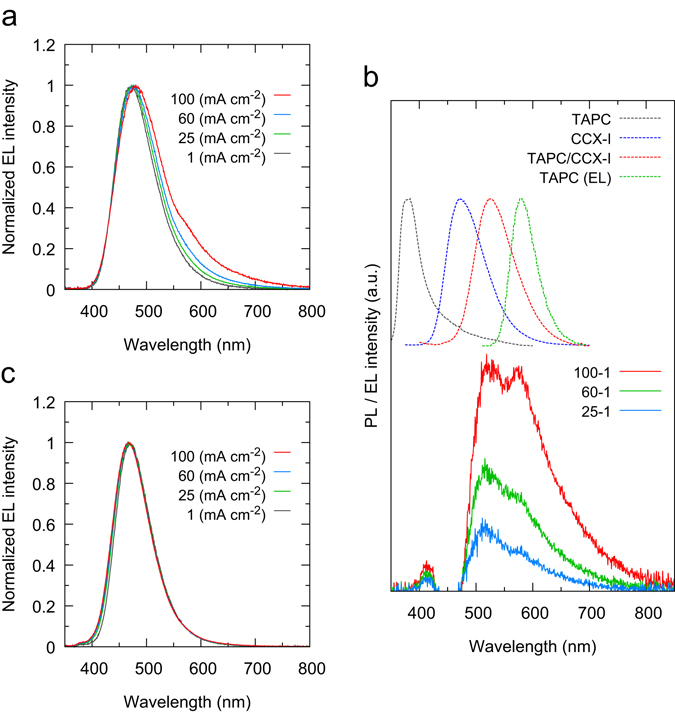
EL spectra of CCX-I based OLEDs. (**a**) EL spectra of CCX-I-6A at *J* values of 1, 25, 60, and 100 mA cm^−2^, with luminance of 88, 961, 1027, and 706 cd m^−2^, respectively. (**b**) Difference of EL spectra of CCX-I-6A (solid lines) and PL spectra of CCX-I, TAPC, and 50 wt% CCX-I:TAPC film together with EL spectrum of TAPC (dashed lines). The difference of spectra was obtained by subtracting the EL spectrum measured at *J* = 1 mA cm^−2^ from those measured at *J* = 25, 60, and 100 mA cm^−2^. (**c**) EL spectra of CCX-I-6B at *J* of 1, 25, 60, and 100 mA cm^−2^, at which the luminance values are 133, 2278, 3783, and 5012 cd m^−2^, respectively.

For blue OLEDs, the choice of appropriate host materials is crucial for effective energy transfer from host to emitting materials. First, we examined bis[2-(diphenylphosphino)phenyl] ether oxide (DPEPO)^44^, a widely used host material for blue TADF-based OLEDs. We fabricated an OLED using CCX-I as the emitting dopant, indium tin oxide (ITO) as an anode, 4,4′-(cyclohexane-1,1-diyl)bis(*N*,*N*-di-*p*-tolylaniline) (TAPC) as a hole-transport layer, 3,3″,5,5″-tetra(pyridin-3-yl)-1,1′:3′,1″-terphenyl (BmPyPhB) as an electron-transport layer, lithium quinolin-8-olate (Liq) an electron-injection layer, and Al as a cathode. The device structure is ITO (50 nm)/TAPC (70 nm)/6 wt% CCX-I:DPEPO (30 nm)/BmPyPhB (40 nm)/Liq (1 nm)/Al (80 nm), termed CCX-I-6A (Device A in Fig. 2a). The CCX-I-6A showed poor device performance: the maximum *η*_EQE_ was 8.2% and drastically decreased at luminance (*L*) greater than 100 cd m^−2^ (x marks in Fig. 2b). Figure 3a shows the EL spectra measured at current densities (*J*) of 1, 25, 60, and 100 mA cm^−2^. The EL intensity in the range of 500–800 nm increased with increasing *J*. Figure 3b in the bottom shows difference of EL spectra obtained by subtracting the EL spectrum measured at *J* = 1 mA cm^−2^ from those measured at *J* = 25, 60, and 100 mA cm^−2^. Two emission bands, with maxima at 520 and 580 nm, appeared in the difference of EL spectra. The latter emission band can be assigned to emission from the TAPC layer^45^. The former emission band may be assigned to emission from an exciplex formed between TAPC and CCX-I; the peak wavelength of 520 nm (2.4 eV) corresponds to the energy difference between the HOMO of TAPC and the LUMO of CCX-I. To verify this exciplex emission, we measured the PL spectra for 50 wt% CCX-I:TAPC, neat CCX-I, and neat TAPC films fabricated by vacuum deposition. The PL spectrum for the 50 wt% CCX-I:TAPC film was clearly different from those for the CCX-I and TAPC neat films, suggesting that the emission from the 50 wt% CCX-I:TAPC film arose from the exciplex formed between CCX-I and TAPC (Fig. 3b). The PL spectrum of the 50 wt% CCX-I:TAPC also agreed well with the emission band at 520 nm in the difference of EL spectra. Meanwhile, the EL spectrum of TAPC agreed well with the emission band at 580 nm in the difference of EL spectra. These observations suggest that emission from the TAPC layer and the exciplex are responsible for the EL emission in the range 500–800 nm, which leads to the poor performance of CCX-I-6A. To prevent the formation of the exciplex, 9,9′-(2,2′-dimethyl-[1,1′-biphenyl]-4,4′-diyl)bis(9*H*-carbazole) (CDBP) was inserted as an interlayer between the TAPC and emissive layers (CCX-I-6B, Device B in Fig. 2a). CDBP has a high T_1_ energy (3.0 eV^46^) and functions as an exciton-blocking layer. In addition, we replaced DPEPO with dibenzo[*b*,*d*]furan-2,8-diylbis(diphenylphosphine oxide) (PPF). PPF shows a higher T_1_ energy than DPEPO and hence, T_1_ excitons are more effectively confined in the emissive layer when PPF is used as a host. To avoid T_1_ energy transfer from the emissive layer to the BmPyPhB layer, a thin PPF layer was inserted as an exciton-blocking layer between the emissive and BmPyPhB layers. The resulting device structure was ITO (50 nm)/TAPC (70 nm)/CDBP (10 nm)/6 wt% CCX-I:PPF (20 nm)/PPF (10 nm)/BmPyPhB (30 nm)/Liq (1 nm)/Al (80 nm). Figure 3c shows the EL spectra of CCX-I-6B measured at *J* = 1, 25, 60, and 100 mA cm^−2^. Unlike CCX-I-6A, no notable changes were observed in the 500–800 nm range. The EL spectrum shows a single emission band with a peak at 468 nm assigned to emission from the 6 wt% CCX-I:PPF layer. Importantly, the *η*_EQE_-*L* characteristics were considerably improved: the maximum *η*_EQE_ of CCX-I-6B was more than twice as high as that of CCX-I-6A, resulting in a *η*_EQE_ of 17.6% (triangles, Fig. 2b). Figure S3a, Supplementary Information, shows the *J*-*V*-*L* characteristic of CCX-I-6B.

By replacing CCX-I with CCX-II in CCX-I-6B, we fabricated CCX-II-based OLEDs with a device structure of ITO (50 nm)/TAPC (70 nm)/CDBP (10 nm)/*X* wt% CCX-II:PPF (20 nm)/PPF (10 nm)/BmPyPhB (30 nm)/Liq (1 nm)/Al (80 nm), where *X* = 6, 10, or 15. Square, rhombus, and filled circle marks in Fig. 2b show the *η*_EQE_-*L* characteristics for the CCX-II-based OLEDs. At *X* = 6, we obtained the maximum *η*_EQE_ of 25.9%, which is the highest value reported for blue TADF-OLEDs to date. The *J*-*V*-*L* characteristics of CCX-II-6B are shown in Figure S3a, Supplementary Information. The peak wavelength of the EL spectra for CCX-II-6B was 471 nm, corresponding to CIE coordinates of (0.15, 0.22) (the left photograph, Fig. 2b). The *η*_EQE_ and colour purity are comparable to those obtained with the blue phosphorescent emitter, FIr6, which has CIE coordinates of (0.14, 0.23)^42^. When using a light out-coupling sheet (CCX-II-6B-OC) we obtained an *η*_EQE_ of 33.3%. The *η*_EQE_ remained at 21.9% even at a high luminance of 1000 cd m^−2^ (open circles and the right photograph, Fig. 2b). Increasing the doping concentration improved the roll-off in the *η*_EQE_-*L* characteristics. For *X* = 15, a maximum power efficiency of 52.5 lm W^−1^ and a current efficiency of 47.5 cd A^−1^ were obtained (Figure S3b, Supplementary Information). These values are high compared with those of other blue TADF-based OLEDs reported to date^10–13, 15–20^. The device characteristics of the CCX-I- and CCX-II-based OLEDs are listed in Table 2.

**Table 2:** OLED performance of devices based on CCX-I and CCX-II.

Devices	EQE_max_^a^ (%)	EQE_100/500/1000_^b^ (%)	PE_max_^c^ (lm W^−1^)	CE_max_^d^ (cd A^−1^)	*λ*_EL_^e^ (nm)	CIE^f^
CCX-I-6B	17.6	13.7/10.9/9.0	24.7	28.3	468	(0.16, 0.21)
CCX-II-6B	25.9	20.2/16.3/14.4	35.9	41.1	471	(0.15, 0.22)
CCX-II-10B	24.5	21.8/19.0/17.0	44.9	44.8	476	(0.16, 0.26)
CCX-II-15B	24.0	22.1/19.7/18.4	52.5	47.5	480	(0.16, 0.29)
CCX-II-6B-OC	33.3	28.9/24.2/21.9	54.4	55.4	469	(0.16, 0.22)

^a^Maximum *η*_EQE_.

^b^ *η*_EQE_ at 100, 500, 1000 cd m^−2^.

^c^Maximum power efficiency.

^d^Maximum current efficiency.

^e^Peak wavelength of EL spectra at 1000 cd m^−2^.

^f^CIE coordinates at 1000 cd m^−2^.

From angular-dependent PL measurements of 6 wt% CCX-I- and CCX-II-doped PPF films, we found that the transition dipole moments of CCX-I and CCX-II were horizontally oriented with respect to the glass substrate, which enhanced their light out-coupling factors. The ratios of the horizontal dipole (*Θ*) for 6 wt% CCX-I- and CCX-II-doped PPF films were determined to be 0.75 and 0.83, which correspond to the order parameters (*S*) of −0.17 and −0.36, respectively (Figure S4a, Supplementary Information). Optical simulations based on these *S* values showed that CCX-I-6B and CCX-II-6B can potentially exhibit *η*_out_ of 25.0% and 29.5%, respectively (Figure S4b,c, Supplementary Information). Using the relation, IQE = *η*_EQE_/*η*_out_, we calculated the IQE values of CCX-I-6B and CCX-II-6B to be 70.4% and 87.8%, respectively. These IQE values are higher than those of conventional fluorescent OLEDs. Thus, the high performance of CCX-I-6B and CCX-II-6B results from efficient TADF and the horizontal orientation of CCX-I and CCX-II molecules.

**Figure 4:**
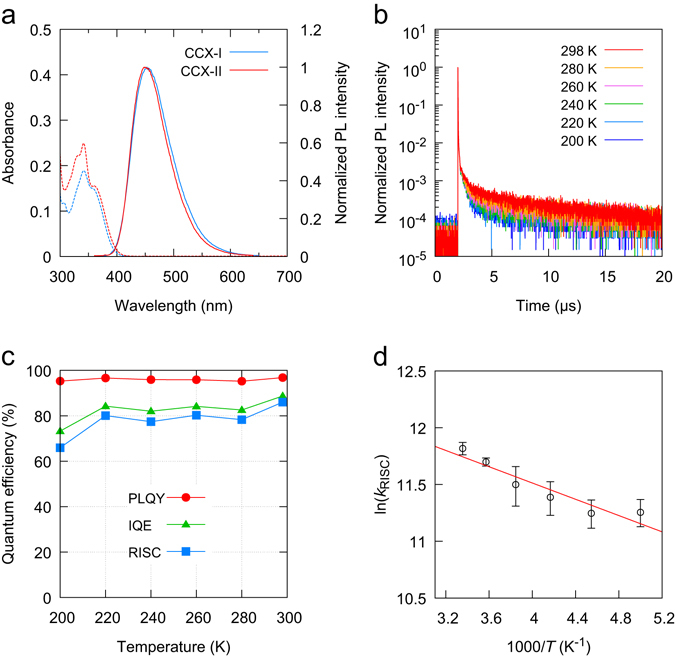
Photophysical properties. (**a**) PL and UV-vis spectra of CCX-I and CCX-II in toluene solution (1.0 × 10^−5 ^M). (**b**) Transient PL decay curves at 200–298 K for a 6 wt% CCX-II:PPF film. (**c**) Temperature dependences of PLQY, RISC efficiency, and IQE for a 6 wt% CCX-II:PPF system. (**d**) Arrhenius plot of the rate constant of RISC for 6 wt% CCX-II:PPF system. A Δ*E*_ST_ of 31 meV was obtained by least-squares fitting (red solid line).

Figure 4a shows ultraviolet-visible (UV-vis) absorption and photoluminescence (PL) spectra of CCX-I and CCX-II in toluene solution (1.0 × 10^−5 ^M). The UV-vis absorption intensity was larger for CCX-II than that for CCX-I, reflecting the greater *f* value of CCX-II than that of CCX-I (Table 1). From the absorption edges (423 nm for both CCX-I and CCX-II), the HOMO-LUMO gaps of CCX-I and CCX-II were confirmed to be sufficiently wide for blue emission. The peak wavelengths of the PL spectra (*λ*_PL_) for CCX-I and CCX-II were 453 and 450 nm, respectively. In dilute toluene solution, CCX-I and CCX-II showed pure blue emission. We also fabricated 6 wt% CCX-I:PPF and CCX-II:PPF thin films by vacuum deposition. The photoluminescence quantum yields (PLQYs) of the 6 wt% CCX-I:PPF and CCX-II:PPF doped films were both nearly 100% (97.2 ± 4% and 104.0 ± 4%, respectively) when CCX-I/CCX-II was directly excited. When PPF was excited, the PLQYs of the molecules decreased to 88.6 ± 4% and 96.8 ± 4%, respectively. The decrease in PLQY suggests that energy loss occurs during the excited energy transfer from PPF to CCX-I/CCX-II. Figure S5, Supplementary Information, shows PL spectra of the 6 wt% CCX-I:PPF and CCX-II:PPF doped films. The *λ*_PL_ of the CCX-I:PPF and CCX-II:PPF doped film were 468 nm and 465 nm, respectively. The emission spectra of CCX-I and CCX-II in the PPF host were red shifted by 15 nm compared with those in toluene solution. When CCX-I/CCX-II was excited, PLQYs of nearly 100% were obtained and no emission from PPF was observed, suggesting that PPF effectively confined the triplet excitons. Figure 4b shows the temperature dependence of transient PL decay curves for the 6 wt% CCX-II:PPF film. In addition to the prompt fluorescence, the long tailed fluorescence was observed. The delayed fluorescence increased with increasing temperature, indicating that it was involved in a thermal activation process. Rate constants for prompt fluorescence, TADF, ISC, and RISC together with contributions from prompt and delayed components to PLQY (*Φ*_p_ and *Φ*_d_, respectively) are reported in Tables S1 and S2, Supplementary Information. Using a method previously reported^40^, we calculated the IQE at 298 K to be 70.6% and 88.7% for CCX-I and CCX-II, respectively. The IQE calculated from the transient PL decays was in good agreement with that obtained from optical simulations (70.4% and 87.8% for CCX-I and CCX-II, respectively). This agreement indicates that excitons are well confined in the emissive layers and losses occurred only in the emitting layer. Figure 4c shows the temperature dependence of the PLQY, RISC efficiency, and IQE of CCX-II doped films. The PLQY of CCX-II is independent of temperature and remained at nearly 100%. The IQE also remained over 80%, suggesting that Δ*E*_ST_ was sufficiently small to induce RISC at room temperature. From an Arrhenius plot of the rate constant of RISC, the Δ*E*_ST_ values of CCX-I and CCX-II were estimated to be 70 and 31 meV, respectively (Fig. 4d and Figure S6, Supplementary Information), which are smaller than that of 4CzIPN (83 meV).

In conclusion, we developed efficient TADF materials, CCX-I and CCX-II with small Δ*E*_ST_ and large *f*. When doped into host matrices, CCX-I and CCX-II showed high PLQYs and blue emission. OLEDs containing CCX-II as an emitting dopant achieved an EQE of 25.9%, which is the highest reported to date among blue TADF-based OLEDs. The OLEDs also showed good colour purity with CIE coordinates of (0.15, 0.22). Further device optimization, using host materials that produce a higher IQE than that of PPF, would allow additional improvements in the performance of these CCX-I- and CCX-II-based OLEDs.

## Methods

### Quantum chemical calculations

The ground states geometries of CCX-I and CCX-II were optimized by DFT calculations. The minimum excited energies of S_1_ and T_1_ were obtained by TD-DFT calculations. All calculations were performed at the PBE0/6-31G(d) level.

### Synthesis and characterization

CCX-I and CCX-II were synthesized, as detailed in Section 1 of the Supplementary Information, with yields of 76% and 100%, respectively. ^1^H and ^13^C nuclear magnetic resonance (NMR) spectra were recorded on a Bruker Avance III 800-MHz spectrometer (800 MHz for ^1^H, 201 MHz for ^13^C). CCX-I and CCX-II were used after purification by temperature-gradient sublimation.

### Device fabrication and measurement of OLED performance

OLEDs with an active area of 4 mm^2^ were fabricated by vacuum deposition at ~10^−5^ Pa on clean ITO-coated glass substrates with a deposition apparatus (SE-4260, ALS Technology, Japan). After fabrication, devices were encapsulated with a desiccant and a glass cap using epoxy glue in a N_2_-filled glove box. The OLED characteristics were measured with a source meter (2400, Keithley, Japan) and an absolute EQE measurement system with an integrating sphere (C9920-12, Hamamatsu Photonics, Japan).

## Electronic supplementary material

Supplementary InfO
