# Supplementary material for: Blue organic light-emitting diodes realizing external quantum efficiency over 25% using thermally activated delayed fluorescence emitters
Source: Sci Rep. 2017 Mar 21;7:284. doi: 10.1038/s41598-017-00368-5 (PMC5428273; doi:10.1038/s41598-017-00368-5)
Supplement: Supplementary file 1 — Supplementary InfO [file 41598_2017_368_MOESM1_ESM.pdf]

Supplementary Information for:

Blue organic light-emitting diodes realizing external quantum efficiency over 25% using thermally activated delayed fluorescence emitters

Takuya Miwa[1], Shosei Kubo[1], Katsuyuki Shizu[1], Takeshi Komino[2], Chihaya Adachi[2,3], Hironori Kaji[1,3*]

[1] Institute for Chemical Research, Kyoto University, Uji, Kyoto 611-0011, Japan

[2] Center for Organic Photonics and Electronics Research (OPERA), Kyushu University, 744 Motooka, Nishi, Fukuoka 819-0395, Japan

[3] JST, ERATO, Adachi Molecular Exciton Engineering Project

[*] kaji@scl.kyoto-u.ac.jp

Contents

1. **Synthesis and characterization (Figure S1, S2)**
2. **OLED performances (Figure S3, S4)**
3. **Optical simulations (Figure S5)**
4. **Photophysical properties (Figure S6, S7, Table S1, S2)**

1. Synthesis and characterization

General procedures

Reactions were performed under an Ar atmosphere. Column chromatography was performed with PSQ 60B silica (Fuji Silysia). ^{1}H NMR and ^{13}C NMR spectra were recorded with the Bruker Avance III 800-MHz spectrometer (800 MHz for ^{1}H and 201 MHz for ^{13}C NMR measurements). Chemical shifts are reported in δ ppm, determined against the signal from residual protons in the deuterated solvents for ^{1}H NMR experiments and solvent peaks for ^{13}C NMR spectra as internal standards. Atmospheric pressure chemical ionization (APCI) mass spectra were measured with a Bruker micrOTOF-Q II (Bruker, Germany). Spectral grade solvents and starting materials were purchased from commercial sources and used without further purification. Respective donor and acceptor units of CCX-I and CCX-II were synthesized following previously reported procedures.[1,2] PPF was also synthesized following previously reported procedures.[3] CCX-I, CCX-II and PPF were used after temperature-gradients sublimation.

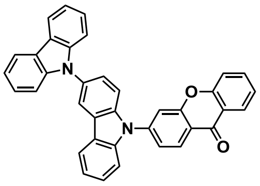

CCX-I

9*H*-3,9′-bicarbazole (BCZ) was prepared according to the method reported in Ref. 1. 3-bromo-9*H*-xanthen-9-one (BXTN) was prepared according to the method reported in Ref. 2. A mixture of BCZ (359 mg, 1.08 mmol), BXTN (297 mg, 1.08 mmol), $Pd_2(dba)_3{\cdot}CHCl_3$ (23.3 mg, 0.0225 mmol), XPhos (40.6 mg, 0.0852 mmol) and *t*-BuONa (158 mg, 1.64 mmol) in toluene (9 mL) was stirred under reflux for 12 h. After cooling to room temperature, the reaction mixture was extracted with $CHCl_3$. The organic layer was washed with H_2O and then dried over anhydrous $MgSO_4$. After filtration and evaporation, the crude product was purified flash chromatography (silica gel, eluent: $CHCl_3$) and recrystallized from toluene to provide CCX-I as a yellow-green solid (429 mg, yield: 76.4%). ^{1}H NMR (800 MHz, $CDCl_3$): δ (ppm) 8.64 (d, J = 8.2 Hz, 1H), 8.43 (dd, J = 7.9 Hz, 1.7 Hz, 1H), 8.32 (d, J = 2.1 Hz, 1H), 8.20 Hz (d, J = 8.2 Hz, 2H), 8.14 (d, J = 8.2 Hz, 1H), 7.87 (d, J = 1.4 Hz, 1H), 7.80 (m, 2H), 7.76 (dd, J = 8.2 Hz, 2.1 Hz, 1H), 7.67 (d, J = 8.2 Hz, 1H), 7.62 (dd, J = 8.2 Hz, 2.1 Hz, 1H), 7.57 (d, J = 8.2 Hz, 1H), 7.54 (dt, J = 8.2 Hz, 6.9 Hz, 1.4 Hz, 1H), 7.47 (dt, J = 8.2 Hz, 6.9 Hz, 1.4 Hz, 1H), 7.43 (m, 4H), 7.39 (t, J = 6.9 Hz, 1H), 7.31 (ddd, 7.7 Hz, 6.0 Hz, 2.1 Hz, 2H); 13C NMR (201 MHz, $CDCl_3$): δ (ppm) 176.39, 157.22, 156.32, 143.43, 141.75, 140.74, 139.11, 135.15, 130.91, 128.91, 127.13, 126.89, 125.92, 125.89, 125.20, 124.44, 123.61, 123.20, 122.17, 122.02, 121.40, 120.83, 120.67, 120.33, 119.74, 119.67, 118.01, 115.28, 111.04, 110.23, 109.73; APCI-MS (*m*/*z*): $[M+H]^+$ calcd. for $C_{37}H_{23}N_2O_2$, 527.1760; found, 527.1991; Elemental analysis calcd. (%) for $C_{37}H_{22}N_2O_2$: C, 84.39; H, 4.21; N, 5.32; found: C, 84.65; H, 4.24; N, 5.29.

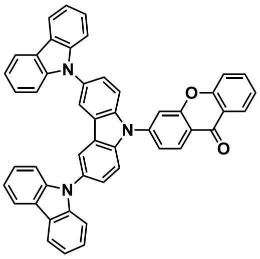

CCX-II

9′*H*-9,3′:6′,9″-tercarbazole (TCZ) was prepared according to the method reported in Ref. 1. A mixture of TCZ (1.00 g, 2.02 mmol), BXTN (553 mg, 2.01 mmol), $Pd_2(dba)_3$·$CHCl_3$ (41.6 mg, 0.0402 mmol), XPhos (76.6 mg, 0.161 mmol) and *t*-BuONa (290 mg, 3.02 mmol) in toluene (20 mL) was stirred under reflux for 23 h. After cooling to room temperature, the reaction mixture was extracted with $CHCl_3$. The organic layer was washed with H_2O and then dried over anhydrous $MgSO_4$. After filtration and evaporation, the crude product was purified flash chromatography (silica gel, eluent: $CHCl_3$) and recrystallized from toluene to provide CCX-II as a yellow-green solid (1.39 g, yield: 99.7%). ^{1}H NMR (800 MHz, $CDCl_3$): δ (ppm) 8.70 (d, 8.9 Hz, 1H), 8.44 (dd, J = 8.2 Hz, 2.1 Hz, 1H), 8.31 (d, J = 2.1 Hz, 2H), 8.17 (d, 8.2 Hz, 4H), 7.95 (d, 2.1 Hz, 1H), 7.86 (d, J = 8.9 Hz, 4H), 7.84 (dd, J = 8.2 Hz, 2.1 Hz, 2H), 7.69 (dd, J = 8.6 Hz, 1.7 Hz, 1H), 7.59 (d, J = 7.6 Hz, 1H), 7.49 (t, J = 8.2 Hz, 1H), 7.41 (m, 8H), 7.30 (m, 4H). ^{13}C NMR (201 MHz, $CDCl_3$): δ (ppm) 176.45, 157.37, 156.42, 143.14, 141.73, 139.88, 135.37, 131.44, 129.27, 127.03, 126.74, 126.07, 124.82, 124.64, 123.34, 122.27, 122.11, 121.08, 120.47, 120.01, 119.95, 118.14, 115.57, 114.54, 109.71. APCI-MS (m/z): $[M]^-$ calcd. for $C_{36}H_{24}N_3$, 691.2260; found, 691.2464; Elemental analysis calcd. (%) for $C_{49}H_{29}N_3O_2$: C, 85.07; H, 4.23; N, 6.07; found: C, 85.03; H, 4.24; N, 6.05.

a

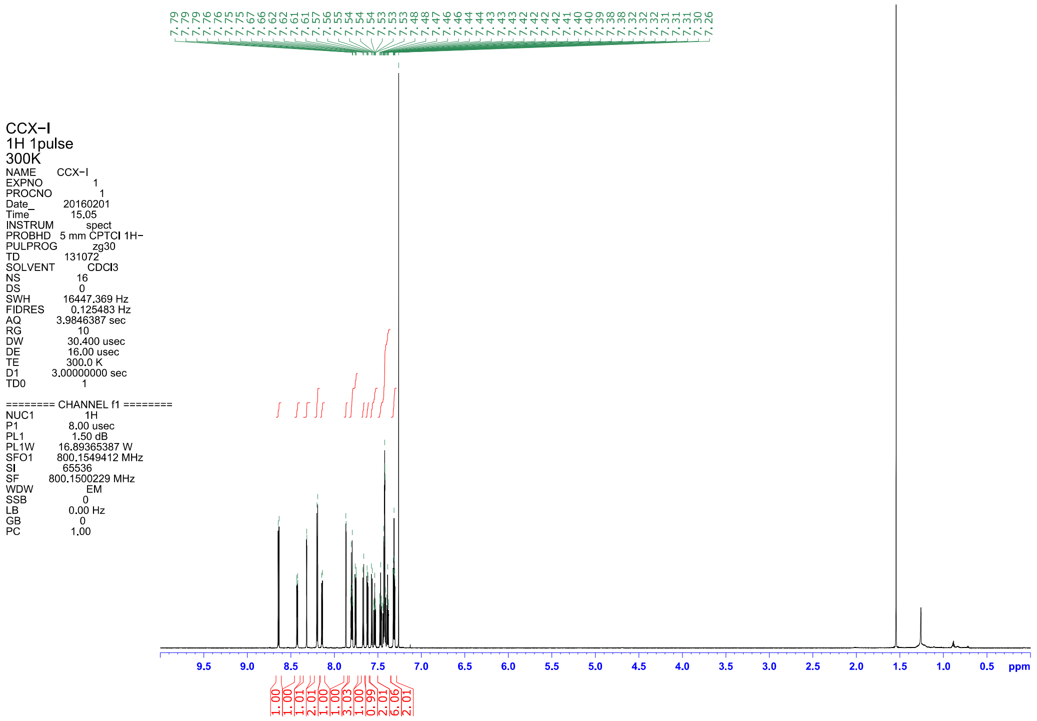

b

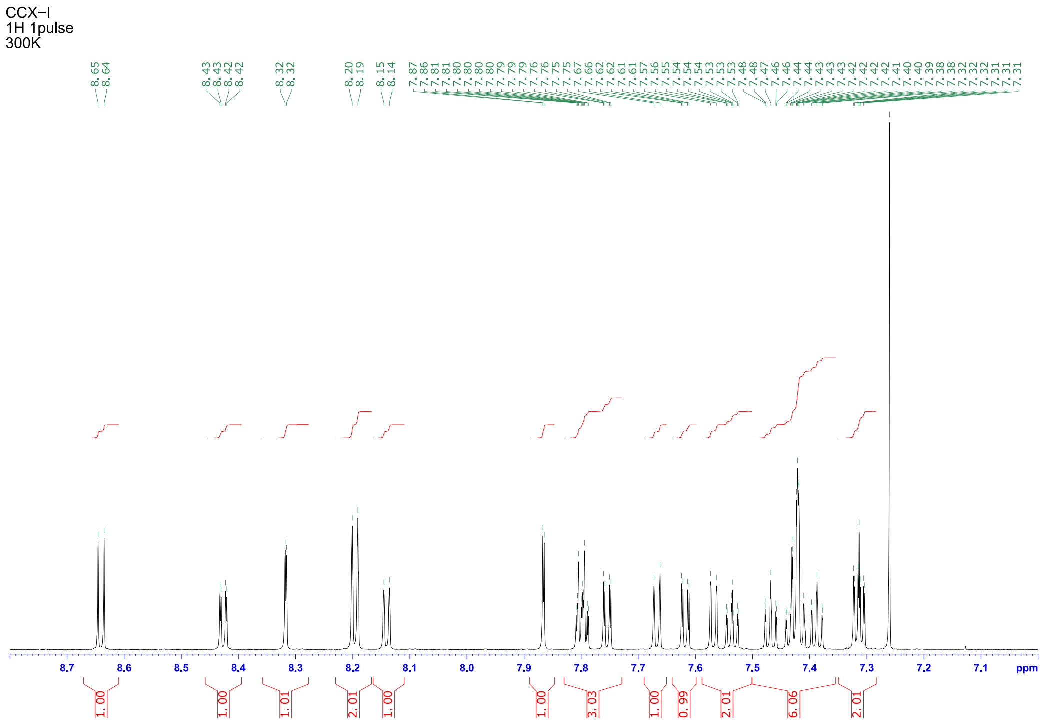

Figure S1 | NMR spectra of CCX-I in $CDCl_3$. a) ^{1}H NMR spectrum. b) Enlarged view of the region 7.0–9.0 ppm. c) ^{13}C NMR spectrum.

c

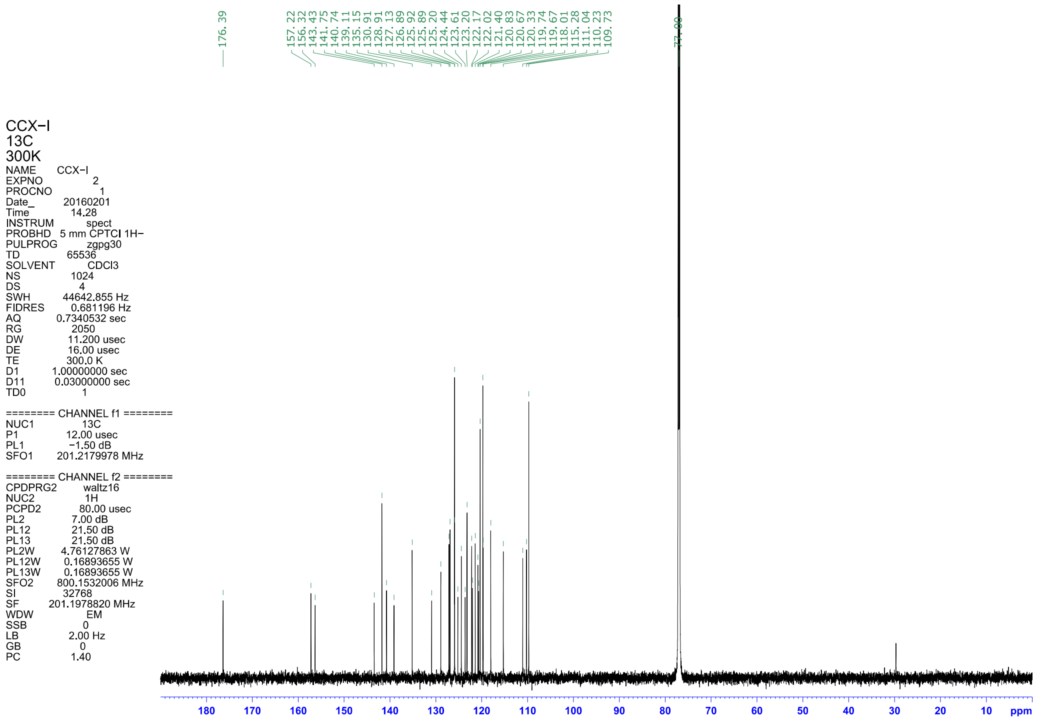

Figure S1 | (contd.)

a

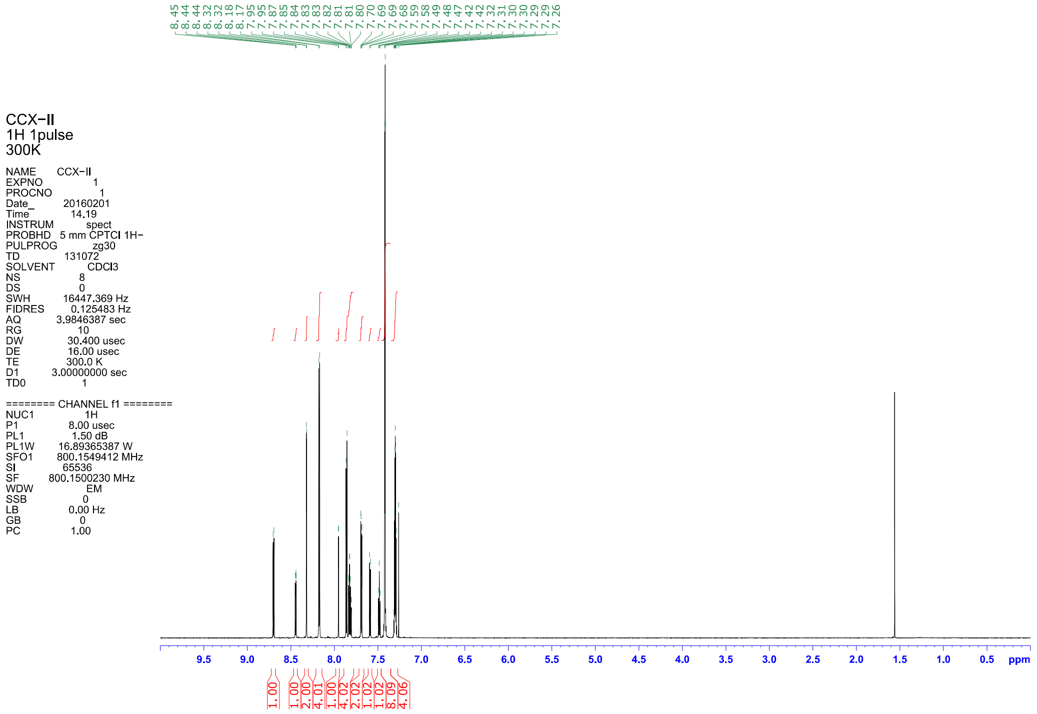

b

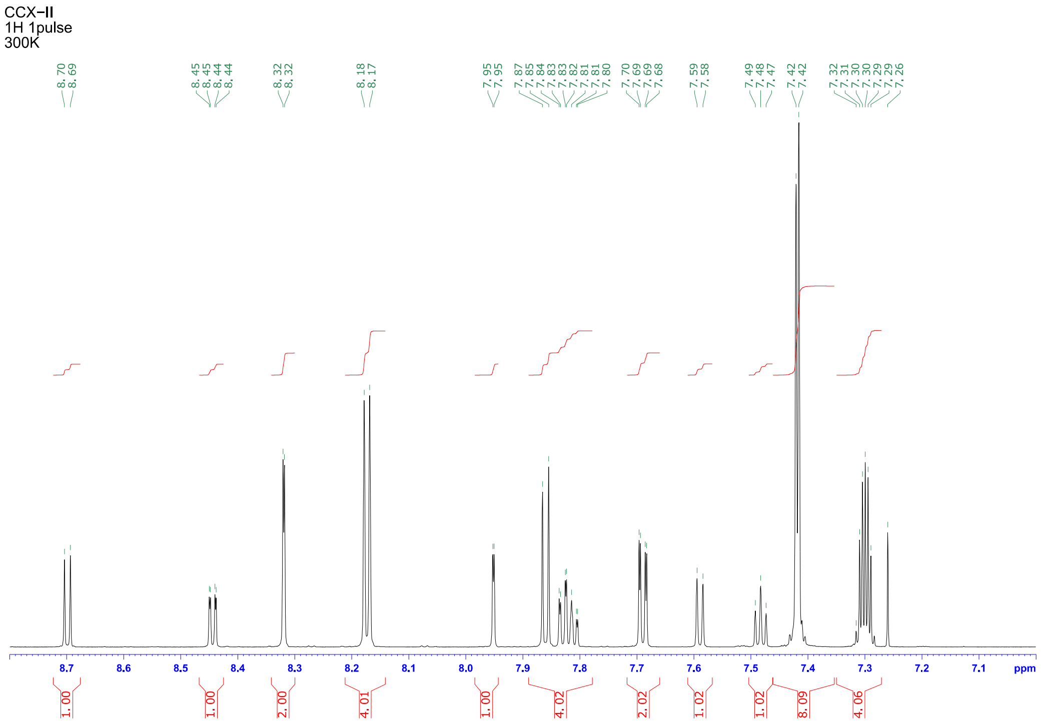

Figure S2 | NMR spectra of CCX-II in $CDCl_3$. a) ^{1}H NMR spectrum. b) Enlarged view of the region 7.0–9.0 ppm. c) ^{13}C NMR spectrum.

c

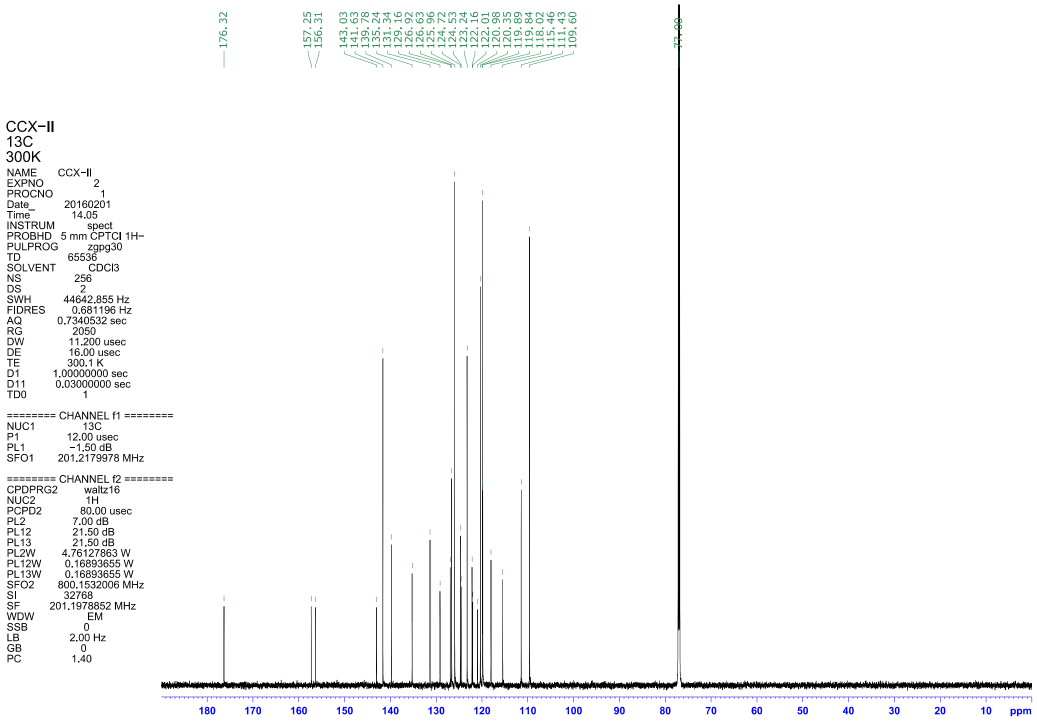

Figure S2 | (contd.)

2. OLED performance

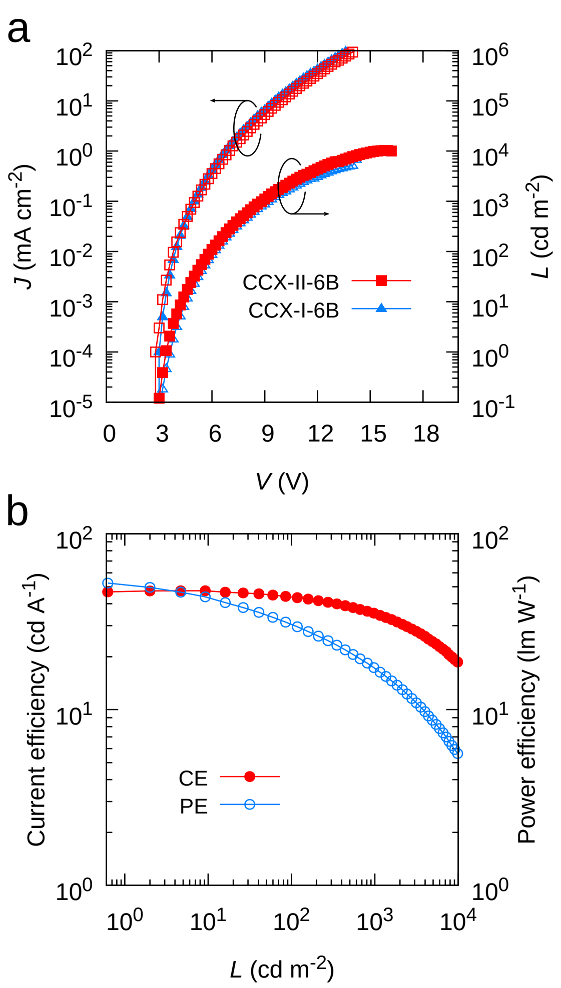

Figure S3 | a) *J-V-L* characteristics for CCX-I-6B and CCX-II-6B devices. b) Current efficiency (CE) and power efficiency (PE) for CCX-II-15B device.

3. Optical simulations

Optical simulations were performed with an opto-electrical device simulator Setfos (Fluxim AG). The optical model used in the simulations featured a glass substrate (0.7 mm) / ITO (50 nm) / TAPC (x nm) / CDBP (10 nm) / emissive layer (EML, 20 nm) / PPF (10 nm) / BmPyPhB (y nm) / Al (80 nm), where x and y are the thicknesses of the hole-transport layer (TAPC) and the electron-transport layer (BmPyPhB), respectively. In the simulations, the emissive dipoles were assumed to be localized in the interface between the CDBP layer and EML.

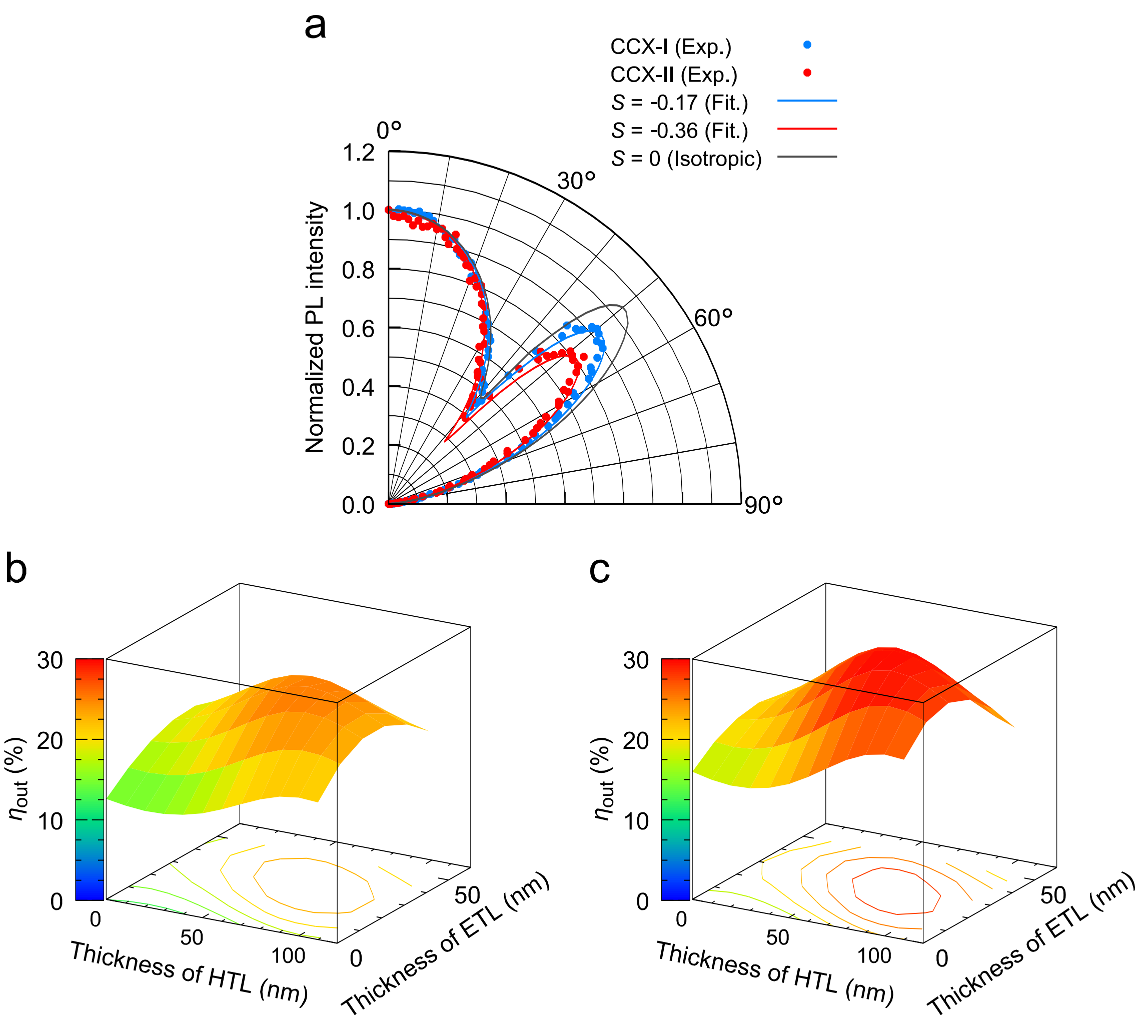

Figure S4 | a) Angular-dependent p-polarized PL intensities for 6 wt% CCX-I: and CCX-II:PPF films. b) and c), Dependence of η_{out} on the thicknesses of the hole-transport layer (HTL) and electron-transport layer (ETL) for CCX-I-6B (b) and CCX-II-6B (c) devices.

4. Photophysical properties

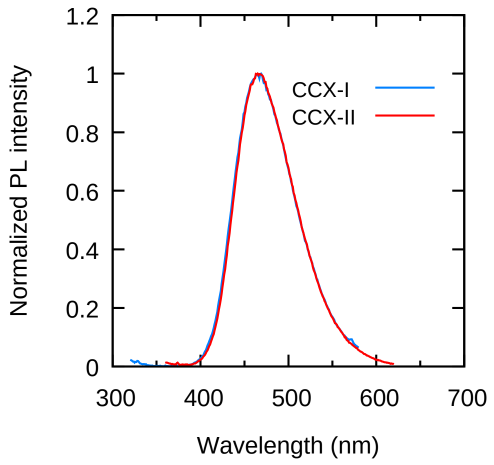

Figure S5 | PL spectra of 6 wt% CCX-I: and CCX-II:PPF films.

Calculation of k_p, k_d, k_{ISC}, and k_{RISC}

From τ_p, τ_d, Φ_{total}, Φ_p and Φ_d, we calculated k_p, k_d, k_{ISC}, and k_{RISC} as previously reported,[4] where τ_p and τ_d are the prompt and delayed fluorescent lifetimes, respectively. Φ_{total}, Φ_p and Φ_d are the PLQY of the total, and prompt and delayed components, respectively. k_p, k_d, k_{ISC}, and k_{RISC} are the rate constants for prompt fluorescence, TADF, ISC, and RISC, respectively. The temperature dependences of the lifetimes, PLQYs and rate constants for 6 wt% CCX-I: and CCX-II:PPF systems are shown in Table S1 and Table S2.

Table S1 | Temperature dependences of lifetimes, PLQYs and rate constants for the 6 wt% CCX-I:PPF system.

Temp. (K)	τ_p (ns)	τ_d (μs)	Φ_{total} (%)	Φ_p (%)	Φ_d (%)	k_p ($\times10^7$ s^{-1})	k_d ($\times10^3$ s^{-1})	k_{ISC} ($\times10^7$ s^{-1})	k_{RISC} ($\times10^3$ s^{-1})
200	8.32	24.9	89.1	76.7	12.5	9.22	4.99	1.28	5.81
220	8.29	18.0	101.8	89.1	12.7	10.7	7.14	1.34	8.15
240	8.10	15.2	83.5	72.4	11.1	8.95	7.34	1.19	8.47
260	7.98	14.5	98.9	82.3	16.9	10.3	11.6	1.74	14.0
280	7.82	14.5	93.4	73.6	19.8	9.41	13.7	1.99	17.4
298	7.77	12.2	88.6	67.7	20.9	8.71	17.2	2.06	22.5

Table S2 | Temperature dependences of lifetimes, PLQYs and rate constants for the 6 wt% CCX-II:PPF system.

Temp. (K)	τ_p (ns)	τ_d (μs)	Φ_{total} (%)	Φ_p (%)	Φ_d (%)	k_p ($\times10^8$ s^{-1})	k_d ($\times10^4$ s^{-1})	k_{ISC} ($\times10^7$ s^{-1})	k_{RISC} ($\times10^4$ s^{-1})
200	7.30	15.2	95.3	86.9	8.3	1.37	6.69	1.79	7.73
220	7.35	15.6	96.6	85.5	11.2	1.36	6.52	1.97	7.66
240	7.18	14.0	95.9	83.8	12.1	1.39	7.35	2.26	8.82
260	7.15	13.2	95.9	81.1	14.7	1.40	7.95	2.64	9.86
280	6.82	10.5	95.2	79.2	16.1	1.47	9.53	3.05	12.0
298	6.82	9.66	96.8	77.0	19.8	1.47	10.5	3.37	13.6

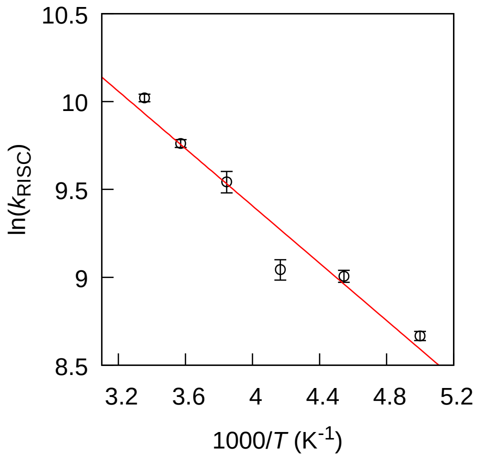

Figure S6 | Arrhenius plot of the rate constant of RISC for the 6 wt% CCX-I:PPF system.

References

1 Hu, D. *et al.* Separation of electrical and optical energy gaps for constructing bipolar organic wide bandgap materials. *Chem. Commun.* **48**, 3015-3017 (2012).

2 Nakanotani, H. *et al.* High-efficiency organic light-emitting diodes with fluorescent emitters. *Nat. Commun.* **5** (2014).

3 Vecchi, P. A., Padmaperuma, A. B., Qiao, H., Sapochak, L. S. & Burrows, P. E. A dibenzofuran-based host material for blue electrophosphorescence. *Org. Lett.* **8**, 4211-4214 (2006).

4 Tao, Y. *et al.* Thermally activated delayed fluorescence materials towards the breakthrough of organoelectronics. *Adv. Mater.* **26**, 7931-7958 (2014).
